# Shared Inflammatory Genetic Susceptibility Underlying Spontaneous Preterm Birth and Periodontitis: A Case–Control Study

**DOI:** 10.3390/jcm14176195

**Published:** 2025-09-02

**Authors:** Joana Couceiro, Carlos Família, José Brito, José João Mendes, Pedro V. Baptista, Alexandra R. Fernandes, Alexandre Quintas

**Affiliations:** 1 UCIBIO—Applied Molecular Biosciences Unit, Department of Life Sciences, NOVA School of Science and Technology, NOVA University Lisbon, 2829-516 Caparica, Portugal; pmvb@fct.unl.pt (P.V.B.); ma.fernandes@fct.unl.pt (A.R.F.); 2 Associate Laboratory i4HB—Institute for Health and Bioeconomy, NOVA School of Science and Technology, NOVA University Lisbon, 2829-516 Caparica, Portugal; 3 Egas Moniz Center for Interdisciplinary Research (CiiEM), Egas Moniz School of Health & Science, 2829-511 Caparica, Portugal; carlosfamilia@egasmoniz.edu.pt (C.F.); jbrito@egasmoniz.edu.pt (J.B.); jmendes@egasmoniz.edu.pt (J.J.M.); aquintas@egasmoniz.edu.pt (A.Q.); 4 Forensic and Psychology Sciences Laboratory Egas Moniz, Campus Universitário Quinta da Granja, 2829-511 Caparica, Portugal

**Keywords:** spontaneous preterm birth, inflammation, periodontitis, genetic polymorphism, *TLR1*, *IL1RN*, *IL6*, *IL6R*, case–control study

## Abstract

**Background**: Preterm birth (PTB) remains the leading cause of neonatal morbidity and mortality worldwide, with approximately two-thirds of cases occurring spontaneously (SPTB), but the etiology is still poorly understood. Chronic inflammatory diseases, such as periodontitis (PD), have been considered SPTB risk factors. However, we hypothesized that SPTB may instead represent a clinical manifestation of a broader genetic predisposition to dysregulated inflammation. Using PD as a model of chronic inflammation, we examined shared genetic susceptibility. **Methods**: In a case–control study (N = 126 Portuguese postpartum women), we screened 56 SNPs in 36 inflammation-related genes. Four functionally plausible variants (*IL1RN* rs4251961, *TLR1* rs5743618, *IL6* rs2069827, and *IL6R* rs4845617) were selected for detailed regression, adjusting for gestational age, floss usage, and an SPTBxPD interaction term. **Results**: *IL1RN* rs4251961 was recessively associated with SPTB risk, consistent with reduced IL-1RA expression linked to this variant. *IL6R* rs4845617 showed a modest protective effect. *TLR1* rs5743618 exhibited the strongest association with the composite “inflammation” phenotype under multiple models, with CC homozygotes showing four-fold increased odds, independent of SPTB/PD co-occurrence. **Conclusions**: This study provides original evidence that shared genetic variants in inflammatory pathways—particularly *TLR1* rs5743618—may underlie susceptibility to SPTB and PD. Our findings suggest a paradigm shift, viewing SPTB as a possible outcome of systemic inflammatory dysregulation rather than merely a consequence of comorbid inflammatory conditions. Future studies should validate this marker in larger cohorts.

## 1. Introduction

Preterm birth (PTB) remains the leading cause of neonatal mortality and morbidity worldwide [1,2]. Although multiple risk factors have been proposed—including inflammatory diseases, maternal comorbidities, and socioeconomic characteristics, among others—its underlying causes are poorly understood. About two-thirds of the cases are spontaneous (SPTB), and the vast majority are idiopathic [3,4]. SPTB is a multifactorial condition resulting from a complex interplay of genetic, environmental, social, and behavioral factors [5], with a substantial contribution from genetic pathways involved in inflammation and immune regulation. Among the many chronic inflammatory conditions considered risk factors for SPTB, periodontitis (PD) has received considerable attention. PD is the second most prevalent oral disease [6], contributes to systemic health issues [7], and is also modulated by genetic predisposition [8]. Although several reviews and meta-analyses have examined the SPTB-PD relationship, findings remain inconsistent—with some reporting significant associations [9,10,11], others only modest links [12,13,14], and still others finding no evidence of interconnection [15,16,17]. A similar pattern emerges in the evaluation of PD treatment as a preventive strategy for PTB: while some reviews suggest a risk reduction [10,18,19], others report insufficient or inconclusive evidence [9,15,16,20,21]. These conflicting data, together with studies challenging the most acceptable direct-translocation hypothesis [10,17,22] and evidence that pregnancy-related periodontal changes are influenced by hormonal and host-specific factors [11,13,23], have led to the proposal of a bidirectional or shared-mechanism model linking SPTB and PD [24]—a model that, to date, remains inconclusive. Given that both conditions have a strong genetic component and are associated with dysregulated inflammatory responses, it is plausible that a shared genetic predisposition to inflammation may underlie individual susceptibility to both conditions. The manifestation of one or the other may then depend on environmental influences and additional host-modifying factors. Indeed, several genes involved in innate immunity and cytokine regulation have been independently associated with either SPTB or PD. However, many of these associations are inconsistent across studies and highly context-dependent, varying by population ancestry, sex, and study design. Importantly, no study has investigated whether these associations with SPTB and PD are present within the same cohort, limiting the assessment of a potentially shared genetic architecture.

A major gap in the literature is the scarcity of original studies examining common genetic risk factors for SPTB and PD. Most available evidence derives from reviews or meta-analyses that often rely on overlapping datasets, potentially introducing bias and limiting insight into shared biological mechanisms. This issue has been highlighted in recent studies that emphasize the need for caution when interpreting systematic reviews on this topic due to data redundancy [19,25]. Targeted genetic association studies focused on candidate inflammation-related genes are, therefore, essential. By enabling well-defined phenotypes and confounder control, these studies provide valuable early insight into the uncovering of shared susceptibility markers in multifactorial diseases like SPTB.

The present study aims to investigate the hypothesis that a shared proinflammatory genetic profile may contribute to susceptibility to both SPTB and PD. We examined selected single nucleotide polymorphisms (SNPs) in genes previously implicated in immune regulation and inflammation, using SPTB as the primary outcome and PD as a model of chronic inflammatory disease. Specifically, we analyzed four SNPs—*TLR1* rs5743618, *IL1RN* rs4251961, and *IL6R* rs4845617—selected based on their biological plausibility and association with inflammation in prior exploratory analysis. By adopting a candidate gene approach and focusing on functionally relevant inflammatory variants, this study provides original data in a largely understudied field. It offers a new perspective on the pathogenesis of SPTB.

## 2. Materials and Methods

### 2.1. Study Design

A case–control study was conducted involving postpartum women recruited from the Puerperium Unit of the Gynecology and Obstetrics Service at Hospital Garcia de Orta (HGO), Portugal, between March 2020 and February 2023. The study was approved by the HGO ethics committee (56/2019), and all participants provided written informed consent. Each participant completed a structured questionnaire assessing sociodemographic and lifestyle variables (e.g., education level, occupation status, smoking, and oral hygiene habits). All procedures were conducted in accordance with the Declaration of Helsinki.

To test the hypothesis of a shared genetic predisposition to inflammation underlying susceptibility to both SPTB or PD, a composite phenotype (“inflammation”) was defined as the presence of either or both conditions. Analyses focused on identifying genetic variants associated with this general inflammatory susceptibility.

### 2.2. Study Population

The sample included 126 postpartum women: 59 with at least one inflammatory condition (SPTB and/or PD) and 67 controls with no diagnosis of these or other inflammatory conditions. Table 1 presents the distribution of participants by group and clinical condition. Due to occasional missing data (i.e., SNP genotypes or covariates), the sample size varied slightly between analyses; the final sample size is reported in each results table.

Clinical diagnosis and blood sample collection were carried out up to 72 h postpartum. SPTB was defined as delivery before 37 completed weeks of gestation following spontaneous labor. Exclusion criteria included multiple pregnancies, preeclampsia, congenital anomalies, and medically indicated PTB. Women with preterm premature rupture of membranes—PPROM—were eligible only if they delivered spontaneously before 37 weeks. PD diagnosis was based on a full-mouth clinical examination. All fully erupted teeth, except for the third molars, dental implants, and retained roots, were evaluated using a manual periodontal North Carolina probe (Hu-Friedy, Chicago, IL, USA). The number of missing teeth, plaque index, gingival recession, periodontal probing depth, and bleeding on probing were recorded at six sites per tooth, and the clinical attachment level was calculated. Furcation involvement was assessed using a 2 N probe (Hu-Friedy; Chicago, IL, USA) in molars and upper first premolars. PD cases were defined according to the American Association of Periodontology consensus. Following the exam, a 2 mL EDTA blood sample was collected, anonymized, and stored at 4 °C (for a maximum of 1 day) until DNA extraction.

Potential confounding factors (e.g., smoking status, oral hygiene habits, education level, and other sociodemographic variables) were evaluated, and those showing significant effects—gestational age and dental floss use—were included as covariates in the logistic regression analyses.

### 2.3. Variant Selection and Genotyping

The initial selection of SNPs was based on an integrative review conducted by our group, which mapped inflammation-related variants potentially involved in SPTB susceptibility [26]. To support the hypothesis of a shared genetic predisposition to inflammation, the panel was expanded to include variants previously associated with PD and other chronic inflammatory or autoimmune conditions. In addition, the STRING database was used to identify additional candidate variants based on protein–protein interaction networks involving known inflammatory-related genes previously associated with SPTB or PD. This approach enabled the inclusion of SNPs that were not highlighted in the initial literature search. In total, 73 SNPs in 36 genes were genotyped using iPLEX Gold technology (Agena Bioscience). In addition to inflammation-related variants, the 73-SNP panel included polymorphisms in genes involved in other biological pathways potentially relevant to both SPTB and PD, such as tissue remodeling (*MMPs*, *TIMPs*), immune activation (*TLR4*, *IFNG*), and growth and angiogenic regulation (*VEGFA*, *TGFB1*, *IGF1*, *IGF1R*). This broader approach allowed us to capture multiple mechanisms that may underlie the shared susceptibility between these conditions. SNPs were excluded if they deviated from the Hardy–Weinberg equilibrium (*p* < 0.05) in controls or had a minor allele frequency (MAF) < 5%, using PLINK v1.9. Preliminary screening using chi-square and Fisher’s exact tests in PLINK was performed to explore the potential contribution of the 73-SNP panel to inflammatory susceptibility. Based on these results and biological plausibility, four SNPs—*IL1RN* rs4251961, *TLR1* rs5743618, *IL6* rs2069827, and *IL6R* rs4845617—were selected for detailed analysis.

### 2.4. Statistical Analysis

Descriptive statistics and group comparisons for sociodemographic and lifestyle variables were performed using R (v4.2.1). Categorical variables were analyzed using the chi-square or Fisher’s exact test, and continuous variables using the Student’s *t*-test. Genetic association analyses were conducted using SPSS (v29.0) via logistic regression, with the inflammation phenotype as the outcome. Initial models estimated raw odds ratios (ORs) under the codominant model. Subsequently, chi-square tests were performed to identify which sociodemographic and lifestyle variables acted as potential confounders in the association between SNPs and the inflammation phenotype. Variables showing significant associations were considered true confounders and included as covariates in the adjusted logistic regression models. Multiple testing corrections using the Bonferroni, Benjamini–Hochberg, and Benjamini–Yekutieli methods were applied in significant associations.

Additionally, the observed statistical power and the sample size required to achieve 80% power were calculated using G*Power (v3.1.9.6). Additive, dominant, and recessive inheritance models were also explored. Finally, to evaluate whether genetic associations persisted independently of the co-occurrence of SPTB and PD, an interaction term (SPTBxPD) was computed and included as a covariate in a secondary adjusted model. This approach aimed to isolate variants related to the broader inflammatory phenotype, regardless of the most severe clinical expression (i.e., women affected by both conditions).

## 3. Results

### 3.1. Demographic and Lifestyle Information of the Control Group and the Inflammation Group

Table 2 presents the demographic and lifestyle data of women in the inflammation group and the control group. The study included 67 controls, aged 31.5 ± 5.53 years, and 59 patients with inflammation, aged 30.9 ± 6.02 years. There were no significant differences in the age parameter between the two groups (*p* > 0.05). In addition, no significant differences were observed between groups for ancestry, education level, occupation status, marital status, number of children, smoking habits, or dietary patterns. Gestational age at delivery was significantly lower in the inflammation group compared to control (*p* < 0.001), and floss usage also differed significantly between groups (*p* = 0.02).

### 3.2. Variant Filtering and Selection

After genotyping, quality control was performed in PLINK: one SNP was excluded due to genotype failure; three additional SNPs were excluded for deviating from the Hardy–Weinberg equilibrium (HWE) (*p* < 0.05) in controls, and 13 variants were excluded due to an MAF below 5%. From the initial panel, 56 SNPs remained for analysis. A preliminary analysis of these 56 SNPs was performed using PLINK to screen for nominal associations (*p* < 0.05) with the inflammation phenotype. Association test outputs are presented in the Supplementary Materials (Supplementary Table S1). Based on these results and biological plausibility, four candidate SNPs—*IL1RN* rs4251961, *TLR1* rs5743618, *IL6* rs2069827, and *IL6R* rs4845617—were selected for further analysis.

### 3.3. Genotype Distribution in Inflammation Group and Controls

Genotype distributions and association results under the codominant model for *IL1RN* rs4251961, *TLR1* rs5743618, *IL6* rs2069827, and *IL6R* rs4845617 are presented below. Table 3 shows the unadjusted logistic regression results, while Table 4 presents the analyses adjusted for gestational age and floss usage, identified as confounding variables through preliminary chi-square analyses of crosstabulations. Secondary exploration analyses under additive, dominant, and recessive inheritance models were also performed and are summarized in Table 5.

Logistic regression results without adjustments for confounders (Table 3) showed that the *TLR1* rs5743618/CC genotype was significantly associated with higher odds of inflammation compared to the AA reference group (OR = 3.65, 95% CI 1.24–10.7, *p* = 0.018). For *IL6R* rs4845617, the AA genotype was associated with reduced odds of inflammation (OR = 0.12, 95% CI 0.02–0.66, *p* = 0.015). Although neither association remained statistically significant after correction for multiple comparisons, the BH-adjusted *p*-value was below 0.1, showing a strong suggestion for this association. Therefore, a sample size estimation was performed for exploratory purposes, indicating that 260 participants would be required to confirm this finding with 80% power. The *IL1RN* rs4251961/CC genotype showed an association with higher odds of inflammation (OR = 3.38, 95% CI 0.99–11.5), although this result was borderline statistically significant (*p* = 0.051). No significant associations were observed for *IL6* rs2069827. After adjusting for gestational age and floss usage (Table 4), the *TLR1* rs5743618/CC genotype remained significantly associated with inflammation (aOR 4.09, 95% CI 1.00–16.69, *p* = 0.049), consistent with the findings from the unadjusted analysis. In contrast, the *IL6R* variant did not remain statistically linked to inflammation. Additionally, the *IL1RN* rs4251961/CC genotype showed significantly increased odds of inflammation compared with the TT reference group (aOR 5.53, 95% CI 1.24–24.74, *p* = 0.025). Exploratory analyses under alternative inheritance models (Table 5) revealed significant associations under the recessive model for *IL1RN* rs4251961 (aOR = 5.09, 95% CI 1.33–19.52, *p* = 0.018) and *TLR1* rs5743618 (aOR = 4.45, 95% CI 1.36–14.58, *p* = 0.014). These associations did not remain statistically significant after correction for multiple testing.

### 3.4. Genetic Association with Inflammation-Adjusted Model Including SPTBxPD Interaction

To test whether specific genetic variants remained associated with the inflammation phenotype independently of the presence of SPTB and PD, we performed a logistic regression analysis adjusted for the interaction term SPTB×PD (Table 6 and Table 7). This model allowed us to control for the possible confounding effect of the most severe clinical manifestation—individuals presenting both conditions—and evaluate genetic contributions to a shared inflammatory susceptibility.

Table 6 presents the logistic regression results under the codominant model for the four selected SNPs, with SPTBxPD as the covariate. Two variants showed a significant correlation with inflammation: The *TLR1* rs5743618 CC genotype was significantly associated with increased odds of inflammation compared to the AA reference (OR = 4.46, 95% CI 1.28–15.58, *p* = 0.019). The *IL6R* rs4845617 AA genotype showed a protective effect (OR = 0.10, 95% CI 0.01–0.95, *p* = 0.044). No significant associations were observed for *IL1RN* rs4251961 or *IL6* rs2069827 in the codominant model. Exploratory analysis under additive and recessive models (Table 7) revealed that *TLR1* rs5743618 remained significant in both the additive (OR = 1.74, 95% CI 0.98–3.10, *p* = 0.060) and recessive (OR = 4.14, 95% CI 1.40–12.21; *p* = 0.010) models. *IL1RN* rs4251961 was also significantly associated with inflammation under the recessive model (OR = 3.74, 95% CI = 1.06–13.21, *p* = 0.041). None of the associations remained significant after multiple testing corrections. These results reinforce the possible role of *TLR1* and *IL1RN* variants in predisposing individuals to a heightened inflammatory state, independent of the simultaneous presence of both clinical conditions.

## 4. Discussion

This study was primarily designed to deepen the understanding of SPTB by exploring whether it might reflect a broader genetic susceptibility to inflammatory dysregulation. SPTB is a complex and multifactorial condition influenced by diverse environmental, social, and biological risk factors. Among the biological correlates, several chronic inflammatory or autoimmune diseases—including PD—have been associated with increased SPTB risk, fostering the widely held notion that these conditions may act as causal risk factors. However, this paradigm has been increasingly challenged by different evidence. Instead, it raises the possibility that SPTB may reflect a broader dysregulation of inflammatory responses rather than being directly caused by specific comorbidities. Thus, this study challenges the conventional paradigm, hypothesizing that SPTB, like other chronic inflammatory conditions, may represent a distinct clinical manifestation of a shared genetic predisposition to dysregulated inflammation, or may arise from a shared predisposition to exaggerated or unresolved inflammation. Rather than setting PD as a causal contributor to SPTB, we used it here as a representative model of chronic inflammatory disease—due to its high prevalence, relative ease of diagnosis, and well-documented (albeit inconclusive) link to SPTB. This integrative approach allowed us to investigate whether common genetic variants involved in inflammation may underlie both conditions, suggesting a shared biological pathway rather than a causal relationship.

From the broader panel of SNPs analyzed, four variants were prioritized for integrative analysis based on their statistical significance and biological plausibility—*IL1RN* rs4251961, *TLR1* rs5743618, *IL6* rs2069827, and *IL6R* rs4845617. The *IL1RN* rs4251961 and *IL6R* rs4845617 variants showed weaker and model-dependent associations. *IL1RN* rs4251961, a functional SNP that reduces IL1RN expression, reached significance only under a recessive model, consistent with previous evidence that low IL-1RA levels may impair the resolution of inflammation and increase the risk of inflammatory diseases [27,28]. *IL6R* rs4845617 was associated with decreased odds of inflammation, suggesting a protective effect potentially mediated through modulation of IL-6 trans-signaling. However, the effects of both variants were sensitive to model assumptions and sample size limitations, underscoring the need for further research. The most robust and consistent association in our study was observed for *TLR1* rs5743618. This nonsynonymous SNP, resulting in the I602S amino acid substitution, has been shown to modulate the intensity of TLR-1-mediated immune responses, likely by impairing surface trafficking [29] and/or inducing conformational changes that attenuate downstream signaling [30]. It also influences the expression of a broad gene network, including genes involved in inflammatory responses [31]. In our cohort, the CC genotype (602S homozygotes) was associated with increased odds of inflammation under multiple genetic models, with statistically significant results retained even after adjustment for confounders and for the SPTBxPD interaction term. This final adjustment was crucial in determining whether the observed association could be attributed to a broader predisposition to inflammation, rather than being solely driven by the co-occurrence of SPTB and PD. By including the SPTBxPD interaction term in the model, we aimed to isolate SNPs associated with inflammatory susceptibility per se, independently of whether both conditions were simultaneously present. The persistence of the *TLR1* rs5743618 association after this adjustment strengthens the evidence that this variant may influence inflammatory imbalance beyond specific disease comorbidity and reinforces the hypothesis of a shared inflammatory genetic background contributing to SPTB.

The biological plausibility of these findings is strengthened by the existing evidence that TLR-1 is a key regulator of immune homeostasis not only through pathogen recognition but also via modulation of apoptosis, epithelial repair, and tissue remodeling. During pregnancy, TLRs are highly expressed in fetal membranes and play essential roles in maintaining maternal–fetal immune tolerance [32]. Dysregulated TLR signaling—whether due to infection or sterile inflammatory triggers—has been implicated in PTB and other adverse pregnancy outcomes. Although rs5743618 has not been previously studied in the context of PTB, its association with other diseases [33] and its demonstrated impact on NfKB signaling [30] and cell apoptosis [34] support its potential relevance in pregnancy-related immune regulation.

Moreover, the impact of this variant extends beyond TLR1 alone, as it has been shown to influence the expression of hundreds of downstream genes and to interact with other SNPs within the *TLR* gene family [35,36]. Such gene–gene interaction may amplify its effect and contribute to a proinflammatory phenotype, particularly in individuals already predisposed to immune imbalance.

Taken together, these findings suggest that rs5743618 may act as a key modulator of host susceptibility to chronic inflammation [37]. In the context of our study, this supports the overarching hypothesis that SPTB may not arise solely from comorbid inflammatory conditions but instead represent one possible clinical manifestation of a shared predisposition to unresolved immune activation. The association observed with *TLR1* rs5743618, therefore, contributes original evidence toward the understanding of SPTB as a condition potentially rooted in systemic inflammatory dysregulation.

To our knowledge, this is the first study to examine SNP associations with both SPTB and PD in the same population, allowing us to explore their potential convergence at the genetic level. The prior literature has focused mainly on reviews and meta-analyses that rely on overlapping datasets, with few original studies directly assessing shared predispositions. By adopting a candidate-gene approach and focusing on functionally relevant variants, this study addresses a critical gap and proposes a novel framework for understanding SPTB as part of a broader inflammatory spectrum. Limitations of this study include the modest sample size, which may limit the power to detect subtle effects. Nonetheless, the robustness of *TLR1* findings across multiple models and adjustments suggests biological and statistical consistency. Future studies with larger cohorts and functional validation will be essential to replicate these associations and clarify mechanistic pathways.

## Figures and Tables

**Table 1:** Distribution of participants by group and condition.

Group	N	SPTB	PD	Both Conditions
Inflammation	59	21 ^1^	26	12
Control	67	0	0	0

N, number of subjects; PD, periodontitis; SPTB, spontaneous preterm birth; ^1^ partial missing data in PD diagnosis for 10 SPTB cases.

**Table 2:** Demographic and lifestyle characteristics of patients with PD and healthy controls.

	Control (N = 67)	Inflammation (N = 59)	*p*-Value
Age			
Mean (SD)	31.5 (5.53)	30.9 (6.02)	0.579
Median [Min, Max]	32.0 [19.0, 43.0]	31.0 [18.0, 43.0]	
European Ancestry			
No	11 (16.4%)	7 (11.9%)	0.611
Yes	56 (83.6%)	52 (88.1%)	
Education Level			
Less than secondary graduation	11 (16.4%)	12 (20.3%)	0.533
Secondary graduation	28 (41.8%)	28 (47.5%)	
Higher education	28 (41.8%)	19 (32.2%)	
Occupation Status			
Unemployed	18 (26.9%)	13 (22.0%)	0.649
Student	1 (1.5%)	2 (3.4%)	
Employed	48 (71.6%)	44 (74.6%)	
Occupation			
No income	12 (17.9%)	7 (11.9%)	0.135
Low income	31 (46.3%)	38 (64.4%)	
High income	24 (35.8%)	14 (23.7%)	
Marital Status			
Unpartnered	25 (37.3%)	29 (49.2%)	0.381
Cohabiting	20 (29.9%)	16 (27.1%)	
Married	22 (32.8%)	14 (23.7%)	
Gestational Age			
<28	0 (0%)	5 (8.5%)	<0.001 *
28–31	0 (0%)	6 (10.2%)	
32–36	0 (0%)	21 (35.6%)	
≥37	67 (100%)	27 (45.8%)	
Previous PTB			
No	63 (94.0%)	52 (88.1%)	0.345
Yes	4 (6.0%)	7 (11.9%)	
Smoker			
No	61 (91.0%)	47 (79.7%)	0.122
Yes	6 (9.0%)	11 (18.6%)	
Missing	0 (0%)	1 (1.7%)	
Ex-smoker			
No	51 (76.1%)	36 (61.0%)	0.119
Yes	16 (23.9%)	22 (37.3%)	
Missing	0 (0%)	1 (1.7%)	
Daily Tooth Brushing Frequency			
Once	7 (10.4%)	8 (13.6%)	0.798
Twice	44 (65.7%)	38 (64.4%)	
Three or more	16 (23.9%)	12 (20.3%)	
Missing	0 (0%)	1 (1.7%)	
Floss Usage			
Never	15 (22.4%)	19 (32.2%)	0.018 *
Occasionally	2 (3.0%)	8 (13.6%)	
One to three times a week	23 (34.3%)	9 (15.3%)	
Everyday	27 (40.3%)	22 (37.3%)	
Missing	0 (0%)	1 (1.7%)	
Dairy Consumption			
Once a week	1 (1.5%)	4 (6.8%)	0.513
Once a day	18 (26.9%)	13 (22.0%)	
Twice a day	34 (50.7%)	29 (49.2%)	
Three times a day	14 (20.9%)	13 (22.0%)	
Cereals Consumption			
Occasionally	2 (3.0%)	3 (5.1%)	0.107
Once a week	7 (10.4%)	0 (0%)	
Once a day	25 (37.3%)	22 (37.3%)	
Twice a day	23 (34.3%)	25 (42.4%)	
Three or more times a day	10 (14.9%)	9 (15.3%)	
Other Autoimmune/Chronic Inflammatory Diseases			
No	61 (91.0%)	52 (88.1%)	1
Yes	6 (9.0%)	6 (10.2%)	
Missing	0 (0%)	1 (1.7%)	
Family History of Chronic Diseases—First Degree Relatives			
No	47 (70.1%)	44 (74.6%)	0.691
Yes	20 (29.9%)	15 (25.4%)	
Early Childhood Feeding			
Unknown	3 (4.5%)	5 (8.5%)	0.692
Formula-fed	12 (17.9%)	11 (18.6%)	
Breast feeding	52 (77.6%)	43 (72.9%)	

N, number of subjects; SD, standard deviation. * Parameter with statistically significant difference between control and PTB groups (*p* < 0.05).

**Table 3:** Genotype distributions and logistic regression results for *IL1RN* rs4251961, *TLR1* rs5743618, *IL6* rs2069827, and *IL6R* rs4845617 in women with inflammation and controls. Only *p*-values < 0.05 were considered for multiple testing correction and power estimation.

SNP	Genotype	Control	Inflammation	OR	95% CI	*p*-Value	*p*-Value ^a^	Sample Size 80% Power
		N (%)	N (%)					
*Il1RN* rs4251961	TT	31 (45.0)	21 (36.2)	1 (ref)				
	TC	29 (43.9)	24 (41.4)	1.20	0.50–2.85	0.688		
	CC	6 (9.0)	13 (22.4)	3.38	0.99–11.5	0.051 ^1^		
*TLR1* rs5743618	AA	29 (43.9)	20 (34.5)	1 (ref)				
	AC	29 (43.9)	18 (31.0)	0.84	0.34–2.04	0.693		
	CC	8 (12.1)	20 (34.5)	3.65	1.24–10.7	**0.018**	0.144(B), 0.072 ^1^ (BH), 0.196(BY)	180
*Il6* rs2069827	GG	54 (81.8)	55 (94.8)	1 (ref)				
	GT	11 (16.7)	3 (5.2)	0.28	0.07–1.17	0.081 ^1^		
	TT	1 (1.5)	0 (0.0)	NA		NA		
*Il6R* rs4845617	GG	21 (31.8)	27 (46.6)	1 (ref)				
	GA	34 (51.5)	29 (50.0)	0.66	0.29–1.50	0.323		
	AA	11 (16.7)	2 (3.4)	0.12	0.02–0.66	**0.015**	0.12(B), 0.072 ^1^ (BH), 0.326(BY)	260

Note: N = 124 women (58 with inflammation, 66 controls) included in this analysis after excluding two cases with missing genotype data. B, Bonferroni correction; BH, Benjamini–Hochberg correction; BY, Benjamini–Yekutieli correction; CI, confidence interval; N, number of subjects; OR, odds ratio; ref, reference genotype; SNP, single nucleotide polymorphism; ^a^ adjusted *p*-value; ^1^ association at the borderline lack of statistical significance (0.05 ≤ *p* < 0.1); the *p*-value in bold indicates statistical significance.

**Table 4:** Genotype distributions and logistic regression results for the association between *IL1RN* rs4251961, *TLR1* rs5743618, *IL6* rs2069827, and *IL6R* rs4845617 with inflammation, adjusted for confounding factors. Only *p*-values < 0.05 were considered for multiple testing correction and power estimation.

SNP	Genotype	Control	Inflammation	aOR	95% CI	*p*-Value	*p*-Value ^a^
		N (%)	N (%)				
*Il1RN* rs4251961	TT	31 (45.0)	20 (35.1)	1 (ref)			
	TC	29 (43.9)	24 (42.1)	1.45	0.44–4.78	0.542	
	CC	6 (9.0)	13 (22.8)	5.53	1.24–24.74	**0.025**	0.20(B), 0.196(BH), 0.679(BY)
*TLR1* rs5743618	AA	29 (43.9)	20 (35.1)	1 (ref)			
	AC	29 (43.9)	18 (31.6)	1.21	0.37–3.99	0.751	
	CC	8 (12.1)	19 (33.3)	4.09	1.00–16.69	**0.049**	0.392(B), 0.196(BH), 0.666(BY)
*Il6* rs2069827	GG	54 (81.8)	54 (94.7)	1 (ref)			
	GT	11 (16.7)	3 (5.3)	0.23	0.02–2.11	0.192	
	TT	1 (1.5)	0 (0.0)	NA	NA	NA	
*Il6R* rs4845617	GG	21 (31.8)	26 (45.6)	1 (ref)			
	GA	34 (51.5)	29 (50.9)	1.41	0.46–4.34	0.550	
	AA	11 (16.7)	2 (3.5)	0.20	0.02–1.97	0.167	

Note: N = 123 women (57 with inflammation, 66 controls) included in this analysis after excluding cases with missing data (two genotypes; one covariate). aOR, odds ratio adjusted for gestational age and floss usage; B, Bonferroni correction; BH, Benjamini–Hochberg correction; BY, Benjamini–Yekutieli correction; CI, confidence interval; N, number of subjects; ref, reference genotype; SNP, single nucleotide polymorphism; ^a^ adjusted *p*-value; the *p*-value in bold indicates statistical significance.

**Table 5:** Exploratory analysis of genetic associations in women with inflammation and controls under additive, dominant, and recessive models, adjusted for confounding factors. Only *p*-values < 0.05 were considered for multiple testing correction.

SNP	Model	aOR	95% CI	*p*-Value	*p*-Value ^a^
*Il1RN* rs4251961	Additive	2.02	0.99–4.10	0.053 ^1^	
	Dominant	2.32	0.81–6.62	0.118	
	Recessive	5.09	1.33–19.52	**0.018**	0.216(B), 0.108(BH), 0.503(BY)
*TLR1* rs5743618	Additive	1.82	0.94–3.52	0.074 ^1^	
	Dominant	1.47	0.53–4.13	0.463	
	Recessive	4.45	1.36–14.58	**0.014**	0.168(B), 0.108(BH), 0.782(BY)
*Il6* rs2069827	Additive	0.20	0.02–1.66	0.135	
	Dominant	0.17	0.02–1.43	0.104	
	Recessive	NA	NA	NA	
*Il6R* rs4845617	Additive	0.71	0.33–1.55	0.390	
	Dominant	1.09	0.38–3.09	0.878	
	Recessive	0.17	0.02–1.48	0.107	

Note: N = 123 women (57 with inflammation, 66 controls) included in this analysis after excluding cases with missing data (two genotypes; one covariate). aOR, odds ratio adjusted for gestational age and floss usage; B, Bonferroni correction; BH, Benjamini–Hochberg correction; BY, Benjamini–Yekutieli correction; CI, confidence interval; SNP, single nucleotide polymorphism; ^a^ adjusted *p*-value; ^1^ association at the borderline lack of statistical significance (0.05 ≤ *p* < 0.1); the *p*-value in bold indicates statistical significance.

**Table 6:** Logistic regression results for the association between *IL1RN* rs4251961, *TLR1* rs5743618, *IL6* rs2069827, and *IL6R* rs4845617 with inflammation, adjusted for the SPTBxPD interaction term. Only *p*-values < 0.05 were considered for multiple testing correction.

SNP	Genotype	Control	Inflammation	aOR	95% CI	*p*-Value	*p*-Value ^a^
		N (%)	N (%)				
*Il1RN* rs4251961	TT	31 (47.0)	16 (33.3)	1 (ref)			
	TC	29 (43.9)	21 (43.8)	0.75	0.27–2.06	0.576	
	CC	6 (9.1)	11 (22.9)	2.89	0.75–11.11	0.122	
*TLR1* rs5743618	AA	29 (43.9)	16 (33.3)	1 (ref)			
	AC	29 (43.9)	16 (33.3)	1.20	0.43–3.37	0.723	
	CC	8 (12.1)	16 (33.3)	4.46	1.28–15.58	**0.019**	0.152(B), 0.152(BH), 0.465(BY)
*Il6* rs2069827	GG	54 (81.8)	46 (95.8)	1 (ref)			
	GT	11 (16.7)	2 (4.2)	0.38	0.07–1.98	0.251	
	TT	1 (1.5)	0 (0.0)	NA	NA	NA	NA
*Il6R* rs4845617	GG	21 (31.8)	22 (45.8)	1 (ref)			
	GA	34 (51.5)	25 (52.1)	0.86	0.33–2.19	0.746	
	AA	11 (16.7)	1 (2.1)	0.10	0.01–0.95	**0.044**	0.352(B), 0.176(BH), 0.538(BY)

Note: N = 114 women (48 with inflammation, 66 controls) included in this analysis after excluding cases with missing data (two genotypes; 10 SPTB cases without PD diagnosis). aOR, odds ratio adjusted for SPTBxPD interaction term (women with both SPTB and PD); B, Bonferroni correction; BH, Benjamini–Hochberg correction; BY, Benjamini–Yekutieli correction; CI, confidence interval; N, number of subjects; ref, reference genotype; SNP, single nucleotide polymorphism; ^a^ adjusted *p*-value; the *p*-value in bold indicates statistical significance.

**Table 7:** Exploratory analysis of genetic associations in women with inflammation and controls, under additive and recessive models, with SPTBxPD as covariate. Only *p*-values < 0.05 were considered for multiple testing correction.

SNP	Model	aOR	95% CI	*p*-Value	*p*-Value ^a^
*Il1RN* rs4251961	Additive	1.38	0.75–2.54	0.304	
	Recessive	3.74	1.06–13.21	**0.041**	0.328(B), 0.096 ^1^ (BH), 0.501(BY)
*TLR1* rs5743618	Additive	1.74	0.98–3.10	0.060	
	Recessive	4.14	1.40–12.21	**0.010**	0.08 ^1^ (B), 0.080 ^1^ (BH), 0.245(BY)
*Il6* rs2069827	Additive	0.27	0.06–1.28	0.098	
	Recessive	NA	NA	NA	
*Il6R* rs4845617	Additive	0.50	0.25–1.01	0.054 ^1^	
	Recessive	0.12	0.01–1.03	0.053 ^1^	

Note: N = 114 women (48 with inflammation, 66 controls) included in this analysis after excluding cases with missing data (two genotypes; 10 SPTB cases without PD diagnosis). aOR, odds ratio adjusted for SPTBxPD interaction term (women with both SPTB and PD); B, Bonferroni correction; BH, Benjamini–Hochberg correction; BY, Benjamini–Yekutieli correction; CI, confidence interval; SNP, single nucleotide polymorphism; ^a^ adjusted *p*-value; ^1^ association at the borderline lack of statistical significance (0.05 ≤ *p* < 0.1); the *p*-value in bold indicates statistical significance.

## Data Availability

The data presented in this study are available on request from the corresponding author due to ethical and privacy restrictions involving human subjects.

## Supplementary Materials

The following supporting information can be downloaded at: https://www.mdpi.com/article/10.3390/jcm14176195/s1, Table S1: Minor allele frequencies and association results for 56 SNPs tested for association with the inflammation phenotype, using PLINK genotypic association test (chi-square).

## Abbreviations

The following abbreviations are used in this manuscript:

HGO	Hospital Garcia de Orta
MAF	Minor allele frequency
PD	Periodontitis
SNPs	Single nucleotide polymorphisms
SPTB	Spontaneous preterm birth

## References

[1] Liu L., Oza S., Hogan D., Chu Y., Perin J., Zhu J., Lawn J.E., Cousens S., Mathers C., Black R.E. (2016). Global, regional, and national causes of under-5 mortality in 2000–2015: An updated systematic analysis with implications for the Sustainable Development Goals. Lancet.

[2] Challis J.R.G., Lye S.J., Gibb W., Whittle W., Patel F., Nadia Alfaidy A. (2001). Understanding preterm labor. Ann. N. Y. Acad. Sci..

[3] Romero R., Dey S.K., Fisher S.J. (2014). Preterm labor: One syndrome, many causes. Science.

[4] Green E.S., Arck P.C. (2020). Pathogenesis of preterm birth: Bidirectional inflammation in mother and fetus. Semin. Immunopathol..

[5] Huri M., Strambi N., Finazzi M., Manciucca G., Catalano G., Seravalli V., Di Tommaso M. (2024). The role of family history of preterm delivery in the individual risk of spontaneous preterm delivery: A case–control study. Arch. Gynecol. Obstet..

[6] World Health Organization (2022). Global Oral Health Status Report: Towards Universal Health Coverage for Oral Health by 2030.

[7] Genco R.J., Sanz M. (2020). Clinical and public health implications of periodontal and systemic diseases: An overview. Periodontol. 2000.

[8] Nibali L., Bayliss-Chapman J., Almofareh S.A., Zhou Y., Divaris K., Vieira A.R. (2019). What Is the Heritability of Periodontitis? A Systematic Review. J. Dent. Res..

[9] Machado V., Ferreira M., Lopes L., Mendes J.J., Botelho J. (2023). Adverse Pregnancy Outcomes and Maternal Periodontal Disease: An Overview on Meta-Analytic and Methodological Quality. J. Clin. Med..

[10] Alnasser B.H., Alkhaldi N.K., Alghamdi W.K., Alghamdi F.T. (2023). The Potential Association Between Periodontal Diseases and Adverse Pregnancy Outcomes in Pregnant Women: A Systematic Review of Randomized Clinical Trials. Cureus.

[11] Chen P., Hong F., Yu X. (2022). Prevalence of periodontal disease in pregnancy: A systematic review and meta-analysis. J. Dent..

[12] Vergnes J.N., Sixou M. (2007). Preterm low birth weight and maternal periodontal status: A meta-analysis. Am. J. Obstet. Gynecol..

[13] Wen X., Fu X., Zhao C., Yang L., Huang R. (2023). The bidirectional relationship between periodontal disease and pregnancy via the interaction of oral microorganisms, hormone and immune response. Front. Microbiol..

[14] Corbella S., Taschieri S., Del Fabbro M., Francetti L., Weinstein R., Ferrazzi E. (2016). Adverse pregnancy outcomes and periodontitis: A systematic review and meta-analysis exploring potential association. Quintessence Int..

[15] Bobetsis Y.A., Graziani F., Gürsoy M., Madianos P.N. (2020). Periodontal disease and adverse pregnancy outcomes. Periodontol. 2000.

[16] Tang L., Chen K. (2024). Association Between Periodontitis and Adverse Pregnancy Outcomes: Two-Sample Mendelian Randomisation Study. Int. Dent. J..

[17] Montoya-Carralero J.M., Ávila-Villasmil R., Sánchez-Pérez A., Jornet-García A., Terrer-Alonso E., Moya-Villaescusa M.J. (2024). Relationship between periodontal disease and preterm birth. A systematic review and meta-analysis. Med. Oral. Patol. Oral. Cir. Bucal.

[18] Merchant A.T., Gupta R.D., Akonde M., Reynolds M., Smith-Warner S., Liu J., Tarannum F., Beck J., Mattison D. (2022). Association of Chlorhexidine Use and Scaling and Root Planing With Birth Outcomes in Pregnant Individuals With Periodontitis: A Systematic Review and Meta-analysis. JAMA Netw. Open.

[19] Arbildo-Vega H.I., Padilla-Cáceres T., Caballero-Apaza L., Cruzado-Oliva F.H., Mamani-Cori V., Cervantes-Alagón S., Vásquez-Rodrigo H., Coronel-Zubiate F.T., Aguirre-Ipenza R., Meza-Málaga J.M. (2024). Effect of Treating Periodontal Disease in Pregnant Women to Reduce the Risk of Preterm Birth and Low Birth Weight: An Umbrella Review. Medicina.

[20] Schwendicke F., Karimbux N., Allareddy V., Gluud C. (2015). Periodontal treatment for preventing adverse pregnancy outcomes: A meta- and trial sequential analysis. PLoS ONE.

[21] Iheozor-Ejiofor Z., Middleton P., Esposito M., Glenny A.-M. (2017). Treating periodontal disease for preventing adverse birth outcomes in pregnant women. Cochrane Database Syst. Rev..

[22] Aagaard K., Ma J., Antony K.M., Ganu R., Petrosino J., Versalovic J. (2014). The placenta harbors a unique microbiome. Sci. Transl. Med..

[23] Carrillo-De-Albornoz A., Figuero E., Herrera D., Bascones-Martínez A. (2010). Gingival changes during pregnancy: II. Influence of hormonal variations on the subgingival biofilm. J. Clin. Periodontol..

[24] Armitage G.C. (2013). Bi-directional relationship between pregnancy and periodontal disease. Periodontol. 2000.

[25] Khan N.S., Craven R., Rafiq A., Rafiq A. (2023). Treatment of periodontal disease in pregnancy for the prevention of adverse pregnancy outcomes: A systematic review of systematic reviews. J. Pak. Med. Assoc..

[26] Couceiro J., Matos I., Mendes J.J., Baptista P.V., Fernandes A.R., Quintas A. (2021). Inflammatory factors, genetic variants, and predisposition for preterm birth. Clin. Genet..

[27] Broderick L., Hoffman H.M. (2022). IL-1 and autoinflammatory disease: Biology, pathogenesis and therapeutic targeting. Nat. Rev. Rheumatol..

[28] Gabay C., Lamacchia C., Palmer G. (2010). IL-1 pathways in inflammation and human diseases. Nat. Rev. Rheumatol..

[29] Johnson C.M., Lyle E.A., Omueti K.O., Stepensky V.A., Yegin O., Alpsoy E., Hamann L., Schumann R.R., Tapping R.I. (2007). Cutting Edge: A Common Polymorphism Impairs Cell Surface Trafficking and Functional Responses of TLR1 but Protects against Leprosy. J. Immunol..

[30] Hawn T.R., Misch E.A., Dunstan S.J., Thwaites G.E., Lan N.T.N., Quy H.T., Chau T.T.H., Rodrigues S., Nachman A., Janer M. (2007). A common human TLR1 polymorphism regulates the innate immune response to lipopeptides. Eur. J. Immunol..

[31] Quach H., Rotival M., Pothlichet J., Loh Y.H.E., Dannemann M., Zidane N., Laval G., Patin E., Harmant C., Lopez M. (2016). Genetic Adaptation and Neandertal Admixture Shaped the Immune System of Human Populations. Cell.

[32] Huebener P., Schwabe R.F. (2013). Regulation of wound healing and organ fibrosis by toll-like receptors. Biochim. Biophys. Acta-Mol. Basis Dis..

[33] Qi H., Sun L., Wu X., Jin Y., Xiao J., Wang S., Shen C., Chu P., Qi Z., Xu F. (2015). Toll-like receptor 1(TLR1) Gene SNP rs5743618 is associated with increased risk for tuberculosis in Han Chinese children. Tuberculosis.

[34] Li X., Jiang S., Tapping R.I. (2010). Toll-like receptor signaling in cell proliferation and survival. Cytokine.

[35] Uciechowski P., Imhoff H., Lange C., Meyer C.G., Browne E.N., Kirsten D.K., Schröder A.K., Schaaf B., Al-Lahham A., Reinert R.R. (2011). Susceptibility to tuberculosis is associated with TLR1 polymorphisms resulting in a lack of TLR1 cell surface expression. J. Leukoc. Biol..

[36] Gutierrez-Castañeda L.D., Acosta C.R., Bustos M.A., García D.K., Bohada D.P., Rodríguez R., Guerrero M.I. (2023). Single Nucleotide Variants in the TLR1, TLR2 and TLR6 Genes: A Case–Control Study in a Colombian Population. Trop. Med. Infect. Dis..

[37] Whitmore L.C., Hook J.S., Philiph A.R., Hilkin B.M., Bing X., Ahn C., Wong H.R., Ferguson P.J., Moreland J.G. (2016). A Common Genetic Variant in TLR1 Enhances Human Neutrophil Priming and Impacts Length of Intensive Care Stay in Pediatric Sepsis. J. Immunol..

