# Supplementary material for: Shared Inflammatory Genetic Susceptibility Underlying Spontaneous Preterm Birth and Periodontitis: A Case–Control Study
Source: J Clin Med. 2025 Sep 2;14(17):6195. doi: 10.3390/jcm14176195 (PMC12428887; doi:10.3390/jcm14176195)
Supplement: Supplementary file 1 [file jcm-14-06195-s001.zip › jcm-3805845-supplementary.pdf]

Table S1. Minor allele frequencies and association results for 56 SNPs tested for association with the inflammation phenotype, using PLINK genotypic association test (chi-square)

SNP	Minor allele	Control	Inflammation	OR	95% CI	*p* value
		Freq (%)	Freq (%)			
***IL6R* rs4845617**	**A**	**0.42**	**0.30**	**0.59**	**0.35-0.99**	**0.045***
KCNN3 rs883319	T	0.18	0.23	1.36	0.73-2.52	0.327
KCNN3 rs1218585	A	0.15	0.24	1.77	0.94-3.35	0.076
KCNN3 rs1218584	G	0.19	0.25	1.42	0.78-2.57	0.251
PTGS2 rs5275	G	0.37	0.41	1.19	0.72-1.98	0.503
PTGS2 rs689466	C	0.16	0.17	1.1	0.56-2.15	0.784
IL10 rs1800872	T	0.34	0.34	1.01	0.6-1.71	0.958
IL10 rs1800871	A	0.34	0.34	1.01	0.6-1.71	0.958
IL10 rs1800896	C	0.37	0.34	0.86	0.51-1.45	0.572
IL1A rs17561	A	0.34	0.33	0.98	0.58-1.65	0.929
IL1A rs1800587	A	0.38	0.36	0.93	0.56-1.56	0.791
IL1B rs1143634	A	0.25	0.24	0.92	0.51-1.63	0.762
IL1B rs1143627	G	0.36	0.47	1.56	0.94-2.59	0.082
IL1B rs16944	A	0.34	0.43	1.46	0.87-2.42	0.148
***IL1RN* rs4251961**	**C**	**0.31**	**0.43**	**1.73**	**1.03-2.9**	**0.038***
TIMP4 rs17035945	T	0.18	0.14	0.77	0.39-1.52	0.452
***TLR1* rs5743618**	**C**	**0.34**	**0.50**	**1.93**	**1.16-3.23**	**0.011***
CXCL8 rs4073	A	0.50	0.49	0.97	0.59-1.59	0.893
NFKB1 rs4648068	G	0.25	0.24	0.92	0.51-1.63	0.762
TLR2 rs4696480	A	0.52	0.48	0.85	0.52-1.4	0.523
TLR2 rs3804099	C	0.48	0.42	0.8	0.49-1.32	0.391
TLR2 rs5743700	T	0.07	0.04	0.61	0.2-1.89	0.391
IL4 rs2243250	T	0.21	0.28	1.47	0.82-2.62	0.191
CD14 rs2569190	A	0.46	0.48	1.08	0.66-1.78	0.759
EBF1 rs2946169	T	0.30	0.31	1.07	0.63-1.84	0.796
TNFA rs1799964	C	0.22	0.25	1.23	0.69-2.21	0.479
TNFA rs1799724	T	0.10	0.09	0.96	0.41-2.22	0.918
TNFA rs1800629	A	0.10	0.19	1.96	0.95-4.04	0.064
TNFA rs361525	A	0.06	0.10	1.78	0.7-4.52	0.218
VEGFA rs2010963	C	0.33	0.34	1.05	0.62-1.77	0.858

SNP	Minor allele	Control	Inflammation	OR	95% CI	*p* value
		Freq (%)	Freq (%)			
IFNGR1 rs11914	C	0.10	0.12	1.15	0.53-2.53	0.721
IFNGR1 rs7749390	G	0.49	0.47	0.93	0.56-1.54	0.767
IFNGR1 rs1327474	C	0.27	0.31	1.2	0.69-2.07	0.523
***IL6* rs2069827**	**T**	**0.10**	**0.03**	**0.24**	**0.07-0.87**	**0.02***
IL6 rs1800796	C	0.10	0.15	1.68	0.78-3.59	0.18
IL6 rs1800795	C	0.30	0.26	0.84	0.48-1.46	0.528
TLR4 rs4986790	G	0.05	0.06	1.34	0.44-4.12	0.602
TLR4 rs7873784	C	0.17	0.18	1.04	0.54-2.0	0.895
MMP8 rs2155052	C	0.15	0.10	0.65	0.3-1.38	0.258
MMP8 rs11225395	A	0.41	0.42	1.04	0.62-1.75	0.869
MMP1 rs7945189	T	0.10	0.07	0.62	0.25-1.54	0.303
MMP3 rs520540	A	0.48	0.37	0.65	0.39-1.08	0.094
MMP3 rs679620	T	0.46	0.36	0.66	0.4-1.1	0.11
IFNG rs2430561	A	0.37	0.36	0.93	0.56-1.55	0.777
IGF1 rs972936	T	0.28	0.26	0.9	0.52-1.57	0.711
IGF1R rs2229765	A	0.42	0.37	0.83	0.5-1.38	0.466
MMP2 rs243865	T	0.19	0.15	0.75	0.39-1.45	0.387
MMP2 rs2285053	T	0.16	0.15	0.92	0.46-1.81	0.801
MMP2 rs11639960	G	0.30	0.20	0.57	0.32-1.02	0.058
TIMP2 rs2277698	T	0.12	0.14	1.24	0.6-2.58	0.562
TIMP2 rs55743137	G	0.22	0.23	1.07	0.59-1.95	0.813
TGFB1 rs1800469	A	0.34	0.27	0.72	0.42-1.25	0.24
MMP9 rs17576	G	0.37	0.25	0.59	0.34-1.02	0.057
TIMP1 rs4898	C	0.40	0.43	1.16	0.7-1.92	0.555
TIMP1 rs2070584	G	0.37	0.43	1.28	0.77-2.12	0.34
TIMP1 rs5906435	T	0.23	0.27	1.24	0.7-2.19	0.466

CI – confidence interval; Freq – minor allele frequency; OR – odds ratio; SNP – single nucleotide polymorphism; * – polymorphism with statistically significant difference between control and inflammation groups ($p < 0.05$); Rows in bold indicates the four SNPs selected for further analysis
